# Biosynthesis of iron oxide nanoparticles using plant extracts and evaluation of their antibacterial activity

**DOI:** 10.1186/s13568-024-01746-9

**Published:** 2024-08-16

**Authors:** Omima Elkhateeb, Mohamed B. Atta, Esawy Mahmoud

**Affiliations:** 1 Food Science and Technology Department, Faculty of Agriculture, Tanta University, Tanta, Egypt; 2 Soil and Water Department, Faculty of Agriculture, Tanta University, Tanta, Egypt

**Keywords:** Biosynthesis iron oxide nanoparticles, Turnip, Moringa, *Escherichia coli*, *Staphylococcus aureus*, Antibacterial agent

## Abstract

The biosynthesis of iron oxide nanoparticles has received increasing attention in the field of food nanotechnology because of their non-toxicity, high efficiency, high antibacterial power, and decontamination features. Therefore, biosynthesis of iron oxide nanoparticles (nFe) was prepared from the leaves of some vegetables, such as cabbage (C) and turnips (T), as well as moringa leaves (M). Alcoholic extracts of these nanoparticles were also tested on *Staphylococcus aureus* and *Escherichia coli* to evaluate their antibacterial activity. The results revealed that the particle sizes of the biosynthesis nanomaterials studied ranged from 12.99 to 22.72 nm, and the particles were spherical, irregular, and surrounded by black color. It also contains many functional groups and minerals. Iron nanoparticles modified with *Moringa oleifera* extract at a concentration of 200 ppm had the highest phenol content compared to other biosynthesis nanoparticles studied. TnFe and MnFe at 200 ppm had a maximum zone of inhibition of 25 mm and 24 mm against *Staphylococcus aureus* and *Escherichia coli*, respectively. While the minimum inhibition zone of 8.0 mm was observed at 25 ppm for nFe against *Escherichia coli*. Therefore, it is recommended to use these extracts of biosynthesis iron oxide nanoparticles as antibacterial agents for stored foods.

**Key points**

- Characterization of prepared biosynthesis iron nanoparticles by spectroscopic analysis.
- Production of biosynthesis iron oxide nanoparticles using various plant extracts with FeSO_4_ solution
- Using the biosynthesis iron oxide nanoparticles as antibacterial agent.

## Introduction

Nanoparticles (NPs) are tiny particles of a metal or substance ranging in size from 1.0 to 100 nm (Shah et al. 2015). They have a large surface area and a small size, which typically results in greater chemical and biological activity compared to large ones (Nel et al. 2006). These properties make them applicable in different fields such as environment, medicine, food, catalysis, electronics, biosensors, and agriculture (Khandel and Shahi 2016; Veeramanikandan et al. 2017). In recent years, attention has been paid to green nanotechnology from various natural sources, such as plants and microorganisms, to produce non-toxic, environmentally friendly, and low-cost nanomaterials compared to chemical and physical methods (Bahrulolum et al. 2021). The synthesis of metal NPs using fungi and bacteria is expensive, and a pollution-free source of insulation must be provided (Kamran et al. 2019). The plant extracts are a potential reducing agent in the synthesis of NPs. The plant extract consists of different bioactive molecules such as phenols, alkaloids, flavonoids, tannins, saponins, vitamins, and amino acids, which reduce ions and stabilize the mineral atom (Amer and Awwad 2021; Shammout and Awwad 2021). Bioactive molecules are characterized by antioxidant effects, many of which have demonstrated antimicrobial activity (Coppo and Marchese 2014).

Phytochemical synthesis is a greenway protocol that has received a lot of attention because it is a new strategy and an alternative to chemical and physical methods (Mittal et al. 2013; Kouhbanani et al. 2019). Cabbage (C), turnip (T), and moringa (M) leaves are non-toxic and biodegradable, making them easy to obtain and environmentally friendly. Moreover, they could be considered waste containing important phytochemicals. Bioactive compounds play a major role in the formation of magnetic nanoparticles and act as capping and stabilizing agents (Archana et al. 2021). The green synthesis of iron oxide nanoparticles (FeNPs) using various plant extracts like *Helianthus annuus* L (Ursache-Oprisan et al. 2011) Sorghum Bran (Njagi et al. 2011), *Hordeum vulgare* and *Rumex acetosa* (Makarov et al. 2014a), *Mimosa pudica* (Niraimathee et al. 2016), *Andean blackberry* (Kumar et al. 2016), *Teucrium polium* (Kouhbanani et al. 2019), and *Moringa oleifera* (Archana et al. 2021) was explored.

Nowadays, interest in biosynthesis nanoparticles as antimicrobial substances has increased for those chemically prepared nanoparticles. Especially since most plants used to make nanoparticles have antimicrobial properties (Kaviya et al. 2011). Add to this that nanoparticle NPs with a large surface area are essential for microbial adhesion and rapid entry into cells (Gurunathan et al. 2014; Yien et al. 2012). Iron oxide nanoparticles represent a promising strategy for combating pathogenicity and resistance to antimicrobial agents, because they may interact with a wide range of bacterial molecules and impede the development of microbes (Zúñiga-Miranda et al. 2023).

Therefore, this study aims to shed some light on the properties of biosynthesis iron oxide nanoparticles (nFe) synthesized from leaf extracts of Moringa (*Moringa oleifera*), Cabbage (*Brassica oleracea*), and Turnip (*Brassica rapa*). The utilization of these extracts as antimicrobial substances was also studied.

## Materials and methods

### Materials

Cabbage (*Brassica oleracea* var. capitata) (C), Moringa (*Moringa oleifera*) (M), or turnip (*Brassica rapa*) (T) were supplied from the Horticulture Department, Faculty of Agriculture, Tanta University. Ferrous sulfate (FeSO_4_) analytical grade was purchased from Sigma-Aldrich (USA). Pure and identity bacterial strains, namely *Staphylococcus aureus* (PTCC 2592) as gram-positive bacteria and *Escherichia coli* (ATCC 25922) as gram-negative bacteria, were kindly supplied as a gift from the Microbiology Department, Faculty of Pharmacy, Kafrelsheikh University. Nutrient agar, MacConkey agar, solvents, and other chemicals used were purchased from El-Gomhoria Company for Chemicals and Drugs, Tanta City, Egypt.

#### Biosynthesis of green iron oxide nanoparticles (nFe)

Biosynthesis iron oxide nanoparticles (nFe) were prepared from cabbage (C), moringa (M), and turnips (T) according to Fahmy et al. (2018), as follows: About 0.5 g of fresh plant leaves were mixed with 1000 mL of distilled water in an electric mixer. The mixture was heated at 70 °C for 45 min, cooled, and filtered through Whatman filter paper. In a 500-ml beaker, place 7.0 ml of a 2 mM FeSO_4_ solution with 10 ml of the aqueous plant extract and stir for 5 min with the pH adjusted above 10. The color of the mixture was changed from translucent yellow to black within 5 min indicating nFe oxide production. The solution was centrifuged for 20 min at 15,000 rpm, at 20 °C. The filtrate was discarded, and the precipitate was rinsed multiple times with distilled water, dried in an electric oven at 60° C and kept at − 4 °C for further analysis.

### Preparation of ethanolic extracts from the synthesized green iron oxide nanoparticles

An alcoholic extract was prepared from the different plants (C, T, and M) with a weight of 0.2 g of nFe in a 15-ml centrifuge tube. Added 10 ml of a 70% (v/v) aqueous ethanolic solution and covered it with a Teflon cover. The mixture was left for 24 h at room temperature. The tube was shaken for 1 min with a vortex, then filtered. Serial dilutions were prepared from the previous extract to reach 200, 100, 50, and 25 ppm.

### Characterization of the synthesized iron oxide nanoparticles

The sizes of nFe, CnFe, MnFe, and TnFe additions were measured using transmission electron microscopy (TEM) using an FEI TECNAI G20 (200KV- LaB6 emitter) microscope at Mansoura University in Egypt. The surface morphology of the analyzed nanomaterials was examined using a scanning electron microscope (SEM) system from JEOL (JSM-7610 F FEG-SEM). The functional groups of the studied nanomaterials were examined using Fourier transform infrared spectroscopy (FTIR). TENSOR 27-Bruker used KBr discs to record the Fourier transform infrared spectra of nanomaterials with wavelengths ranging from 400 to 4000 cm^−1^. The minerals of the examined nanomaterials were identified using X-ray difraction (XRD). A GNR X-ray Diffractometer (APD 2000 PRO) was used to analyse the samples, and diffraction peaks were identified between 2θ = 15◦ and 2θ = 75◦.

### Total phenol content

Biosynthesis iron nanoparticles were combined with Folin-Ciocalteu’s phenol solution, and then 2 ml of Na_2_ CO_3_ (7.5% w/v) was added. Following that, the test tubes were wrapped in aluminum foil, shaken, and incubated for two hours. The absorbance at the wavelength of 765 nm was measured as a blue color, as indicated for phenolic substances. The total phenol content was reported in mg gallic acid equivalents per g of extract (AOAC 2000).

### Determination the efficiency of green nFe oxide as antimicrobial agent

Antibacterial activity for nFe oxide extract against pathogenic *Escherichia coli* (ATCC 25922) and *Staphylococcus aureus* (PTCC 2592) was assessed in triplicate using the penetration diffusion test. Therefore, the growth inhibition zone was in vitro determined according to Masoumi and Esmaeili (2020) as follows: 100 µl of bacterial suspension (10^8^ cfu ml^−1^) was spread over a solid nutrient agar medium on Petri dishes using a sterile glass rod. The 5.0- mm-diameter wells were performed on the surface of nutrient agar under aseptic conditions using a stainless steel aid. Exactly 50 µl of ethanolic extract nFe oxide containing 25, 50, 100, and 200 ppm were placed in these wells. Then the Petri dishes were incubated at 37 °C for 24 h and the diameter of the inhibitory zone (the clear zone free from bacteria growth in mm) was measured as an indication of the antimicrobial activity of nFe oxide. At the same time, blanking was carried out by using 70% (v/v) ethanol free from nFe.

### Statistical analysis

All obtained data were analyzed statistically using DSAASTAT version 1.101 software; five replicas were utilized for the statistical evaluation of variance (ANOVA). The Duncan’s Multiple Range Test (DMRT) was used to compare treatments with a statistical significance level of *P* < 0.05.

## Results

**Fig. 1:**
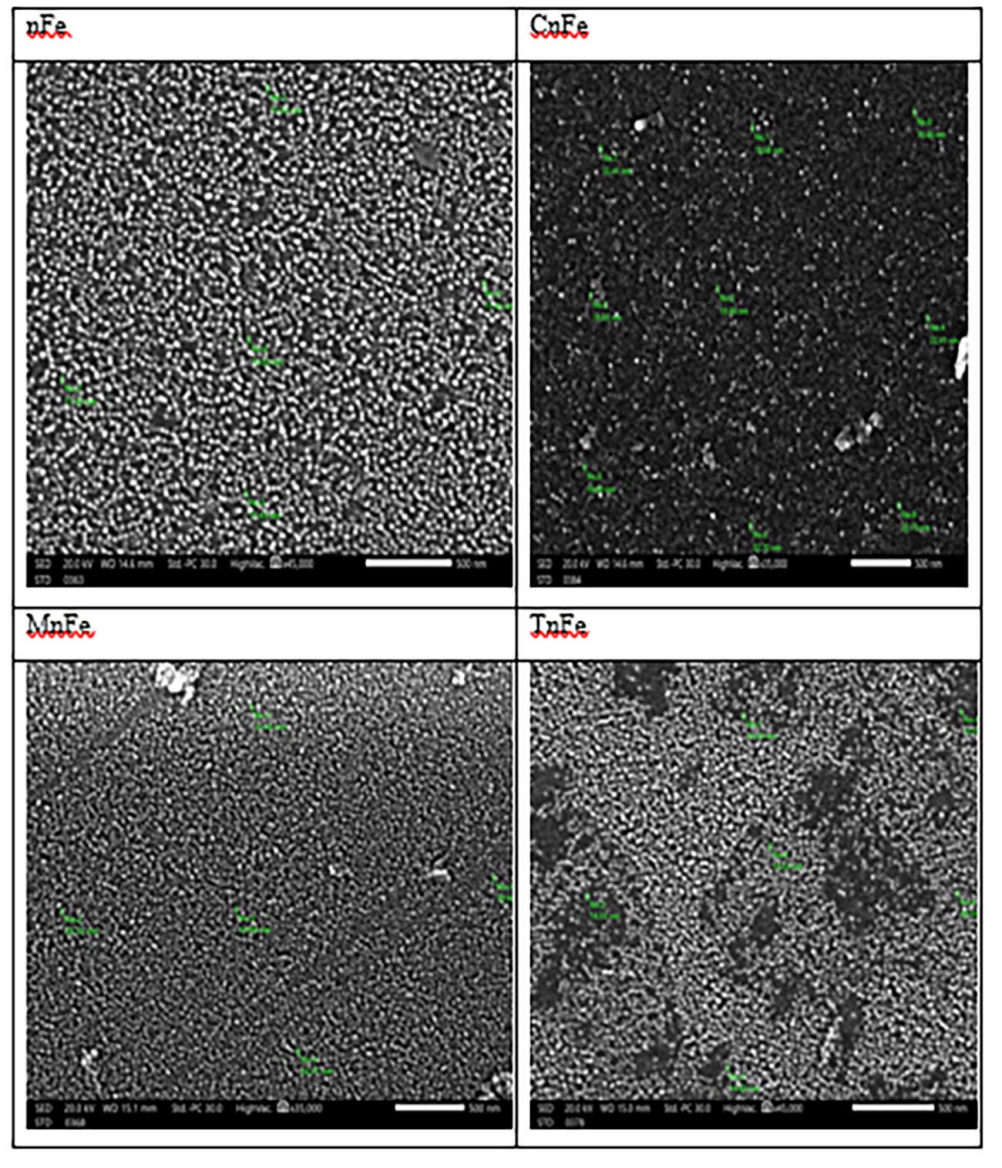
Scanning electron microscopy (SEM) images for different green nanomagnetic (Fe_2_O_3_)

As shown in Fig. 1, scanning electron microscopy (SEM) analysis showed that the synthesized nFe oxide had a spherical shape, nano-rods, and some agglomeration of nanoparticles; the particle size ranged from 14.53 to 17.93 nm. The particle sizes of the biosynthesis nanomaterials investigated ranged from 12.99 to 22.72 nm, and the particles had spherical and irregular forms. However, CnFe particles were more often spherical in shape and surrounded by black color than other nanoparticles. Agglomeration in CnFe, MnFe, and TnFe is absent.

**Fig. 2:**
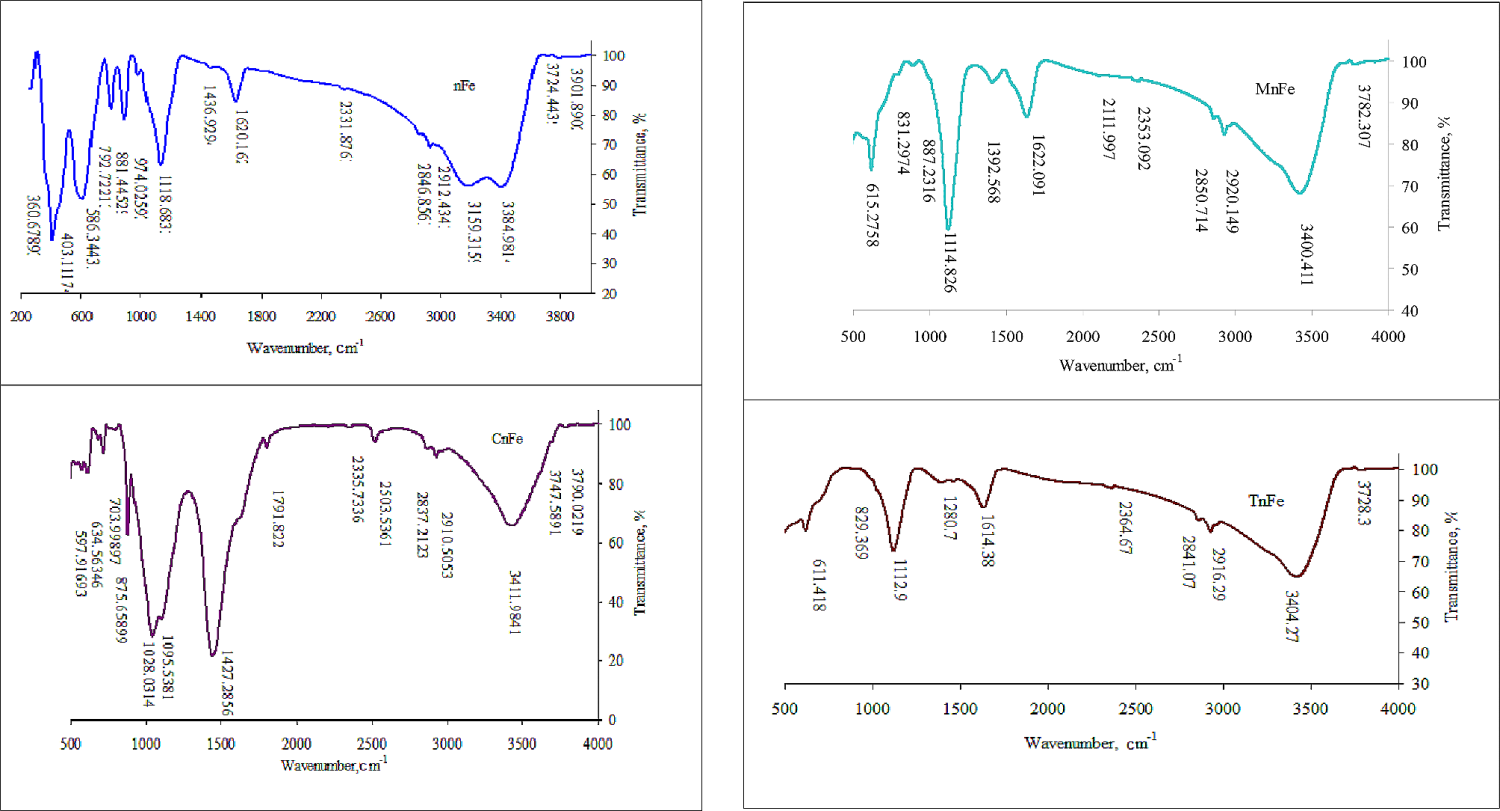
Fourier transform infrared spectroscopy (FTIR) images for different green nanomagnetic (Fe_2_O_3_)

Figure 2 shows the FTIR of the studied nanoparticles containing several peaks, such as peaks at 634 cm^−1^, 611 cm^−1^, 597 cm^−1^, 586 cm^−1^, 403 cm^−1^, and 360 cm^−1^. The peaks appeared at 3782 cm^−1^ in MnFe, 3728 cm^−1^ in TnFe, 3776 and 3426 cm^−1^ in CnFe, and 3384 cm^−1^ in nFe. The bands at 1620 cm^−1^, 1622 cm^−1^, 1630 cm^−1^, and 2916 cm^−1^ appeared in MnFe and TnFe, but 2516 cm^−1^ and 1436 cm^−1^ appeared in CnFe. The peaks at 1114 and 1112 cm^−1^ in MnFe and TnFe represent the symmetric C-O stretching vibrations.

**Fig. 3:**
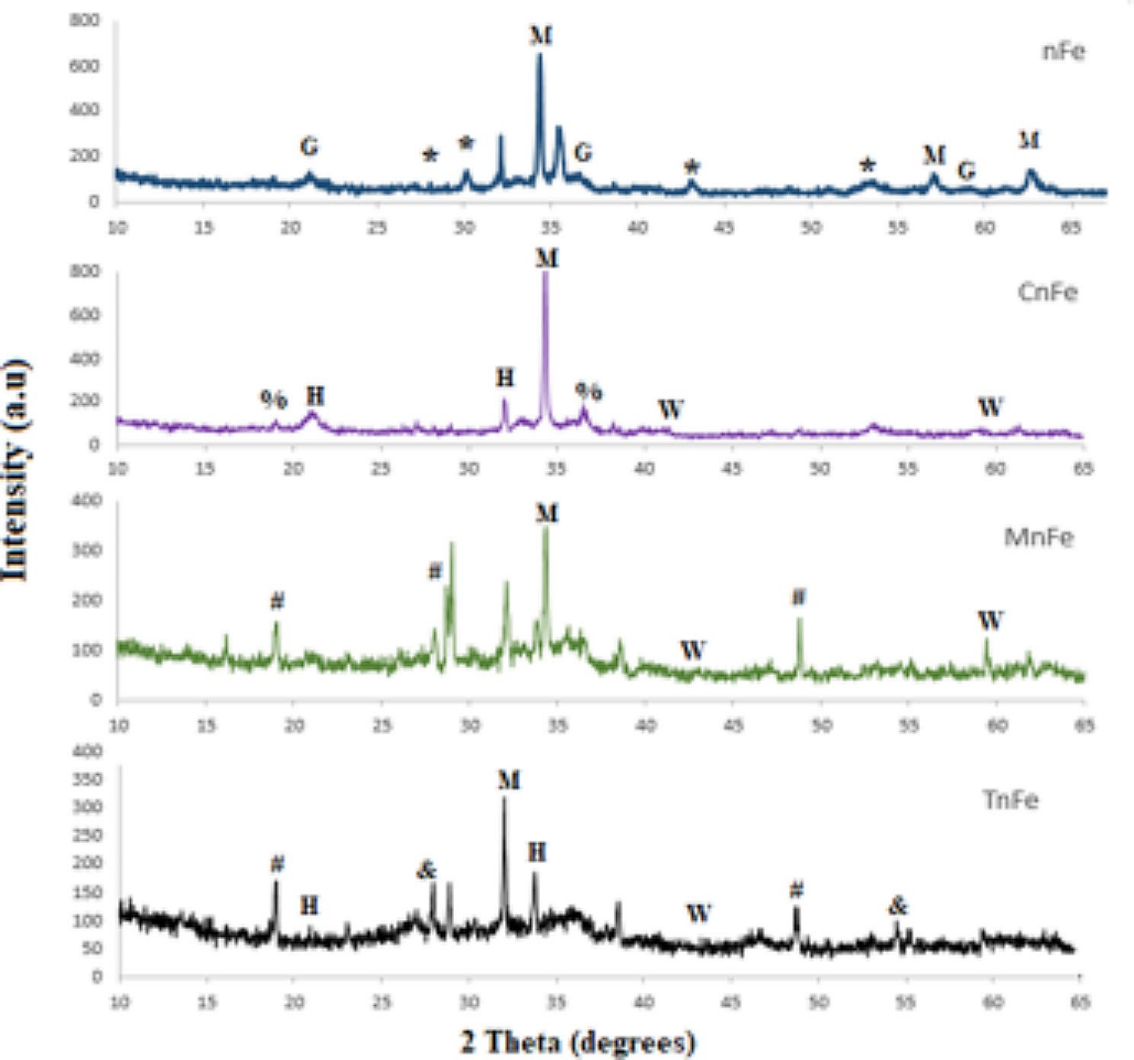
X-ray diffraction (XRD) spectrum of nFe; (M is Magnetite; G is Geothite; and * is Therandite); CnFe; (M is Magnetite; H is Hematite; W is Wustite; and % is HK 2 NO 6 S 2); MnFe; (M is Magnetite; W is Wustite; and # is C 4 H 7 NO 3 ); and TnFe; (M is Magnetite; H is Hematite; W is Wustite; & is Sodium Carbonate Hydrate and # is C 4 H 7 NO 3)

The X-ray diffraction (XRD) spectra of nFe, CnFe, MnFe, and TnFe are shown in Fig. 3. Sharp peaks in nFe indicate the presence of magnetite (ϒ-Fe_2_O_3_), goethite (FeO_2_), and therandite (H_2_Na_2_S). While the peaks in CnFe are indicative of goethite (FeO_2_), hematite (α-Fe_2_O_3_), wustite (FeO), and sulfonate (HK_2_NO_6_S_2_). In MnFe and TnFe, the peaks indicate the presence of magnetite (Fe_2_O_3_), goethite (FeO_2_), wustite (FeO), and organic compounds (C_4_H_7_NO_3_).

The total phenolic content differed significantly among the green nanoparticles studied at a concentration of 200 ppm (Table 1). The total phenol content increased from 8.15 for nFe to 10.24 mg GA/g in nFe treated with moringa extract, an increase of 25.64%. The total phenolic extract compounds in the mayonnaise sample increased from 10.29 mg GA/g for nFe to 11.83 mg GA/g for MnFe, an increase of 15.53%.

**Table 1:** Effect of green nanomaterials on total phenolic concentrations at 200 ppm and mayonnaise extract (50 g)

Treaments	Total phenolic compounds (mg GA/g extract)	Total phenolic extract compounds in the mayonnaise sample (mg GA/g extract)
nFe	8.15^d^	10.24^d^
CnFe	8.56^c^	11.31^c^
TnFe	9.18^b^	11.53^b^
MnFe	10.24^a^	11.83^a^
F-test	**	**
LSD (*p* < 0.05)	4.25	4.48
LSD (*p* < 0.01)	6.18	6.52

**Means with different superscripts (capital letters in the same column and small letters in the same row) are significantly different at (*p* ≤ 0.05) (4.25, 4.48) and (*p* < 0.01) (6.18, 6.52)

Referring to Table 2, results show that the green nanoparticles investigated effectively inhibited the growth of pathogenic bacteria with varying efficiency. MnFe has the highest significant (*p* < 0.05) antibacterial activity against *E. coli* among the other studied nanomaterials. However, TnFe has similar activity against *S. aureus* among the other studied nanomaterials. The antimicrobial activity of nFe oxide increases as the concentration increases. Whereas both TnFe and MnFe extracts at 200 ppm have a maximum inhibition zone of 25 mM and 24 mM against *S. aureus* and *E. coli*, respectively. While the minimum inhibition zone of 8.0 mm was observed for nFe oxide against *E. coli*. In our study, green iron oxide nanoparticles were more effective in inhibiting gram-positive bacteria than gram-negative ones.

**Table 2:** Impact of green nanomaterials as antimicrobial activity agent

Treatments	Concentrations, ppm	Diameter of inhibition zones in mm	
		*Staphylococcus aureus* (Gram positive bacteria)	*Escherichia Coli* (Gram negative bacteria)
Control (Ethanoic 70%)	-	5.0	5.0
nFe	25	8.33^h^	7.00^g^
	50	15.00^de^	12.67^f^
	100	17.67^bc^	18.00^cd^
	200	24.00^a^	19.00^c^
CnFe	25	9.00^h^	8.67^g^
	50	12.00^fg^	16.00^de^
	100	15.33^cde^	17.67^cd^
	200	17.33^bcd^	20.00^bc^
TnFe	25	10.33 ^gh^	9.00^g^
	50	14.33^ef^	13.33^f^
	100	18.67^b^	18.00^cd^
	200	25.00^a^	22.00^ab^
MnFe	25	11.67^g^	13.33^f^
	50	16.33^bcde^	15.00^ef^
	100	18.00^b^	20.00^bc^
	200	22.67^a^	24.00^a^
F-test		**	**
LSD (*p* < 0.05)		2.36	2.59
LSD (*p* < 0.01)		3.18	3.48

**Means with different superscripts (capital letters in the same column and small letters in the same row) are significantly different at (*p* ≤ 0.05) (2.36, 2.59) and (*p* < 0.01) (2.59, 3.48)

## Discussion

In this study, CnFe particles were more often spherical in shape and surrounded by black than other nanoparticles, which suggests that iron sulphide (FeS) was formed as a result of the interaction between iron and the SH-group present in cabbage. The absence of agglomeration in CnFe, MnFe, and TnFe is due to the fact that the polyphenols in the extract reduce the agglomeration of nFe oxide, which improves their reactivity (Makarov et al. 2014b; Naseem and Farrukh 2015).

Peaks at 634 cm^−1^, 611 cm^−1^, 597 cm^−1^, 586 cm^−1^, 403 cm^−1^, and 360 cm^−1^ in the studied nanoparticles were all assigned to the Fe-O stretching and Fe-O-Fe bridging stretching patterns, which indicate the formation of iron oxide nanoparticles (Pallela et al. 2019). Similarly, Mihir and Siddhivinayak (2015) confirmed the formation of iron oxide with Fe-O vibration at 576 nm by FTIR. The O-H stretching vibrations were assigned to bands at 3782 cm^−1^ in MnFe, 3728 cm^−1^ in TnFe, 3776 and 3426 cm^−1^ in CnFe, and 3384 cm^−1^ in nFe. The C-H and H-C = O stretching vibrations were ascribed to the bands at 2920 and 2916 cm^−1^ in MnFe and TnFe (Nakamoto 2009). The S-H and S = O stretching vibrations were assigned peaks at 2516 cm^−1^ and 1436 cm^−1^ in CnFe (Socrates 2004). The peaks at 1114 and 1112 cm^−1^ in MnFe and TnFe represent the symmetric C-O stretching vibrations. The bands at 1620 cm^−1^, 1622, and 1630 cm^−1^ are due to the bending vibration of H_2_O in nFe or may be due to N-H bond stretching vibrations in MnFe and TnFe, which are present in biomolecules as primary amine functional groups. The presence of these bands was the result of phytochemicals found in the leaf extracts of cabbage, moringa, and turnip (Reddy et al. 2020). The observation generally confirms the presence of organic compounds (such as proteins, cardiac glycosides, phenolic compounds, sugar, and flavonoids) in cabbage, moringa, and turnip leaf extracts, which act as stabilizers and reducing agents for nFe oxide. These results are consistent with those of Moustafa and Al Din (2017).

According to Mahdavi et al. (2013), the findings show that the spinel phase structure of hematite, magnetite, and goethite is consistent with the XRD standard for magnetic iron oxide nanoparticles. Magnetite is a very common iron oxide (Fe_3_O_4_) in the studied nanoparticles. Similar to Ali et al. (2017), who the observed that the common phase iron oxide is precipitating magnetite (Fe_3_O_4_) nanoparticles (35–45 nm) at pH 10 during synthesized iron oxide nanoparticles with ferromagnetic behavior.

**Fig. 4:**
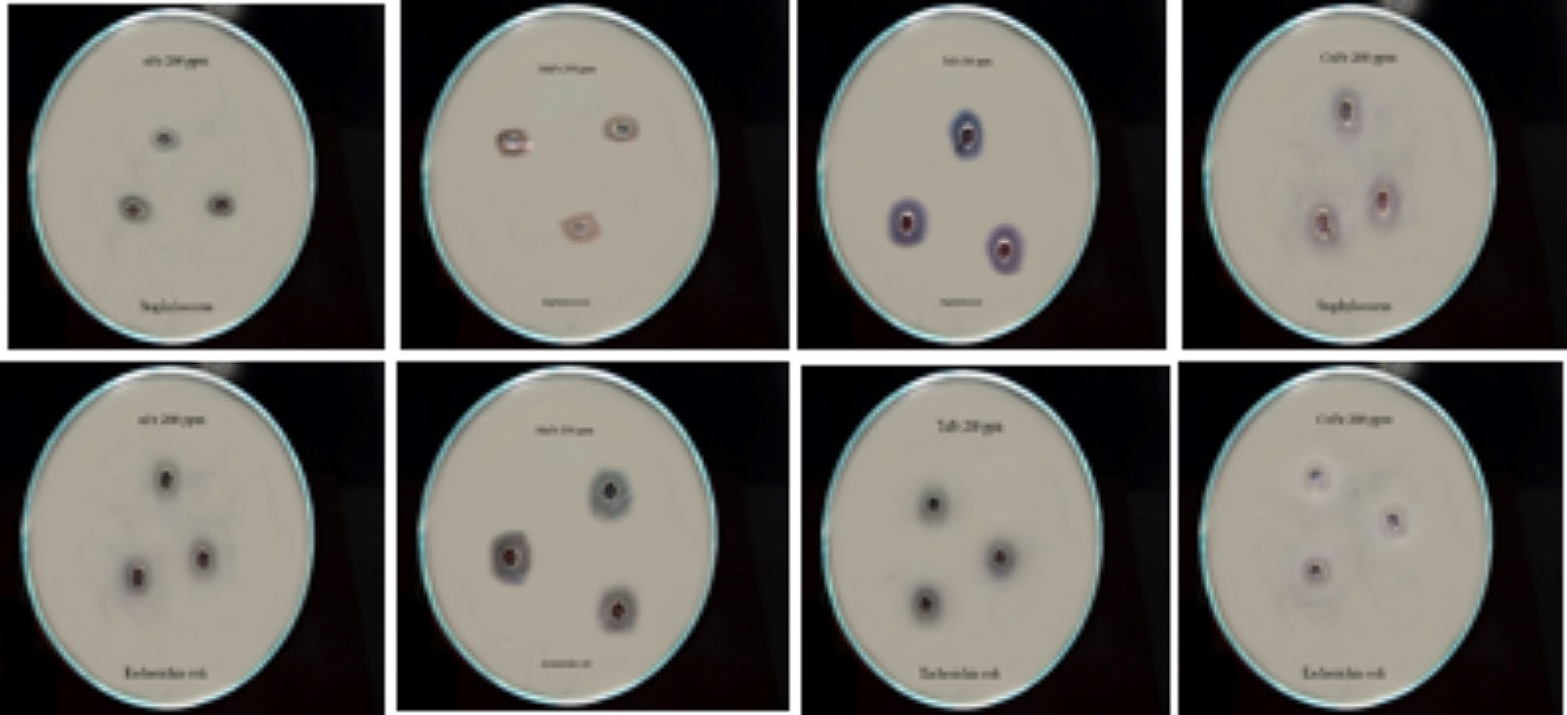
Effect green nanoparticles investigated at 200 ppm as antimicrobial activity agent (as:* Escherichia coli* and *Staphylococcus aureus*)

The intensity peaks at 2ϴ = 19.28° and 36.43° in the CnFe and 2ϴ = 37.26° and 48.84° in each MnFe and TnFe were recognized in Fig. 4 as organic chemicals contained in the leaf extracts of these plants as confirmed by SEM and FTIR. Similarly, they corresponded to the XRD patterns of Fe NPs synthesized with green tea extract.

MnFe has the highest content of phenols compared to other green nanoparticles. This is due to the plant extracts used in synthesized iron oxide nanoparticles, which contain phenolic substances, especially *Moringa oleifera* extract. Emmanuel et al. (2014) and Patel et al. (2014) discovered that aqueous extracts of *Moringa oleifera* contain phytochemicals such as flavanoids, terpenoids, and various polyphenols.

The studied biosynthesis nanoparticles showed an effective effect in inhibiting the growth of pathogenic bacteria that were tested for *Escherichia coli* and *Staphylococcus aureus*. Similar results were obtained by Vitta et al. (2020), who stated that the biosynthesis of FeNPs using *Eucalyptus robusta* extract had a high potential for antibacterial activity against *Pseudomonas aeruginosa*,* Escherichia coli*,* Staphylococcus aureus*, and *Bacillus subtilis.* Al-Karagoly et al. (2022) showed that nFe oxide had excellent antibacterial activity against *E. coli* with inhibition zones of 12.34 ± 0.58 mm and *S. aureus* with inhibition zones of 11.52 ± 0.58 mm, respectively. Aksu Demirezen et al. (2022) found that green iron oxide nanoparticles (nFe) had the largest inhibitory zone against *S. aureus* (24.27 ± 0.12 mm) when compared to four traditional antibiotics, including cefotaxime (≥ 23 mm), tetracycline (≥ 19 mm), gentamicin (≥ 15 mm), and cefoxitin (≥ 22 mm). The antibacterial impact of nFe was more effective against *S. aureus* bacteria than against *E. coli*.

The main mechanism by which green iron oxide nanoparticles inhibit bacteria growth is related to the oxidative stress generated by reactive oxygen substances (ROS), including hydrogen peroxide (H_2_O_2_), hydroxyl radicals (^−^OH), superoxide radicals (O^−2^), and singlet oxygen (^1^O_2_), which cause damage to proteins and DNA in bacteria, leading to cell death (Mohapatra and Anand 2010; Tran et al. 2010). In the present study, the iron oxide (FeO) confirmed by spectroscopic analyses could be the source that creates ROS species that lead to inhibition of the studied bacteria. A similar process has been described by Kim et al. (2007), where hydrogen peroxide (H_2_O_2_) reacts with Fe^2^ through the Fenton reaction and forms hydroxyl radicals, which are known to be damaging to biological molecules. Lee et al. (2008) demonstrated that zero-valent iron nanoparticles (NZVI) with a size ranging from 10 to 80 nm are able to penetrate *Escherichia coli* membranes and interact with intracellular oxygen, leading to oxidative stress and cell membrane destruction. Nanoparticle shapes play an important role in damaging bacterial cells to varying degrees through interactions with periplasmic enzymes (Cha et al. 2015). In our study, green iron oxide nanoparticles were more effective in inhibiting gram-positive bacteria than gram-negative ones. This may be explained by the fact that gram-positive bacteria have a thin layer of peptidoglycan in their cell wall, which provides efficient contact between the organism and the nanoparticles. This is in contrast to gram-negative bacteria, which have a thick layer of peptidoglycan on their cell walls that impedes the contact between nanoparticles and living matter in the cell, making them less effective (Uchenna et al. 2018).

The results of the study showed that the properties of green iron oxide are characterized by functional groups (such as Fe-O, H-C = O, S-H, C-O, and N-H) and minerals (such as magnetite, goethite, hematite, wustite, and sulfonate). MnFe has the highest content of phenols compared to other green nanoparticles. The biosynthesis nanoparticles investigated effectively inhibited the growth of pathogenic bacteria with varying efficiency. MnFe has the highest significant (*p* < 0.05) antibacterial activity against for *E. coli* and TnFe against *S. aureus* among the other nFe oxides. The antibacterial activity against *E. coli* and *S. aureus* was higher when biosynthesis nanoparticles investigated were added at 200 ppm than at the levels at 25 and 100 ppm. The result recommends the application of 200 ppm of MnFe or TnFe to achieve high antibacterial activity. However, further research studies are highly needed to assess the application of green nFe oxides in stored foods.

## Data Availability

The datasets used and analyzed during the current study are available from the corresponding author on reasonable request.
